# Chorion-derived extracellular matrix hydrogel and fibronectin surface coatings show similar beneficial effects on endothelialization of expanded polytetrafluorethylene vascular grafts

**DOI:** 10.1016/j.mtbio.2022.100262

**Published:** 2022-04-16

**Authors:** Sabrina Rohringer, Karl H. Schneider, Gabriela Eder, Pia Hager, Marjan Enayati, Barbara Kapeller, Herbert Kiss, Ursula Windberger, Bruno K. Podesser, Helga Bergmeister

**Affiliations:** a Medical University of Vienna, Center for Biomedical Research, Währinger Gürtel 18-20, 1090, Vienna, Austria; b Ludwig Boltzmann Institute for Cardiovascular Research, Währinger Gürtel 18-20, 1090, Vienna, Austria; c Medical University of Vienna, Department of Obstetrics and Gynaecology, Division of Obstetrics and Feto-Maternal Medicine, Währinger Gürtel 18-20, 1090, Vienna, Austria; d Austrian Cluster for Tissue Regeneration, Vienna, Austria

**Keywords:** Small diameter vascular grafts, ePTFE, Endothelialization, Human placental chorionic hydrogel

## Abstract

The endothelium plays an important regulatory role for cardiovascular homeostasis. Rapid endothelialization of small diameter vascular grafts (SDVGs) is crucial to ensure long-term patency. Here, we assessed a human placental chorionic extracellular matrix hydrogel (hpcECM-gel) as coating material and compared it to human fibronectin in-vitro. hpcECM-gels were produced from placental chorion by decellularization and enzymatic digestion. Human umbilical vein endothelial cells (HUVECs) were seeded to non-, fibronectin- or hpcECM-gel-coated expanded polytetrafluorethylene (ePTFE) SDVGs. Coating efficiency as well as endothelial cell proliferation, migration and adhesion studies on grafts were performed. hpcECM-gel depicted high collagen and glycosaminoglycan content and neglectable DNA amounts. Laminin and fibronectin were both retained in the hpcECM-gel after the decellularization process. HUVEC as well as endothelial progenitor cell attachment were both significantly enhanced on hpcECM-gel coated grafts. HUVECs seeded to hpcECM-gel depicted significantly higher platelet endothelial cell adhesion molecule-1 (PECAM-1) expression in the perinuclear region. Cell retention to flow was enhanced on fibronectin and hpcECM-gel coated grafts. Since hpcECM-gel induced a significantly higher endothelial cell adhesion to ePTFE than fibronectin, it represents a possible alternative for SDVG modification to improve endothelialization.

## Graphical abstract

**Figure:**
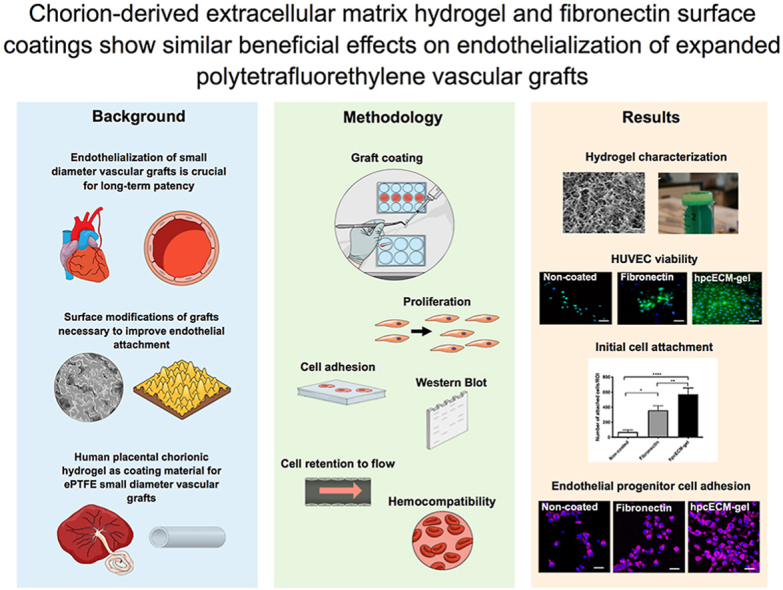

## 1 Introduction

Cardiovascular disease is the major cause for mortality in Western countries [1]. Advanced vascular disease often requires the substitution of non-functional blood vessels. However, the development of grafts with an inner diameter of less than 6 ​mm which depict satisfying patency rates has remained a major challenge. Although several recent pre-clinical studies with biodegradable synthetic polymers [[2], [3], [4], [5], [6], [7], [8], [9], [10], [11]], as well as natural extra-cellular matrices [[12], [13], [14], [15], [16], [17]], or hybrids [[18], [19], [20]] yield hope for future clinical use, the goal to produce a long-term patent small diameter vascular graft (SDVG) remains unmet so far. Therefore, in this study, a home-made coating material derived from human placental chorionic plate was tested for its beneficial effect on endothelialization of ePTFE vascular grafts.

The current state-of-the-art material for synthetic SDVGs is expanded polytetrafluorethylene. In clinics, ePTFE grafts are used in patients for whom autologous vascular grafting is not possible due to limited availability, vascular disease, or infeasible invasive harvest procedure. However, ePTFE grafts have considerably lower patency rates than autologous implants. Besides infection, atherosclerosis progression, intimal hyperplasia and thrombosis formation are major causes for graft failure [21]. These limitations restrict the use of ePTFE grafts in challenging approaches such as in coronary bypass procedure [22]. Endothelial cells can inhibit intimal hyperplasia [23] and provide an anti-thrombotic environment in the lumen of blood vessels [24]. Therefore, the endothelialization of vascular grafts is highly advantageous to improve their patency rates [21].

Various methods such as growth factor delivery [25,26], adhesion-associated molecule delivery [27,28] or other surface modifications [29,30] were applied to improve endothelialization of PTFE grafts. Very convincing long-term patency rates in clinical observations were achieved by in-vitro cell-seeded ePTFE grafts with fibrinolytically inhibited fibrin glue as coating material [31,32].

Since the 1980s, several studies have indicated the beneficial effect of fibronectin protein on the endothelialization of ePTFE vascular grafts [26,33,34]. Furthermore, it has been shown that primary patency rates improved significantly by populating fibrin-coated ePTFE grafts with autologous vein endothelial cells prior to implantation [31,32,35]. Despite the promising outcomes, there is no fibronectin available for clinical use [36,37]. This is due to the lack of good manufacturing practice (GMP) in fibronectin production [31]. Assmann et al. (2013) found elevated levels of intimal hyperplasia in fibronectin-coated decellularized aortic vessel grafts compared to non-coated implants already after 8 weeks of implantation in rats [38]. The use of pure fibronectin for graft coating should be reconsidered since subendothelial matrix remodeling towards a fibronectin-rich basal lamina by endothelial cell fibronectin deposition induces atherosclerotic plaque formation [39].

ECM hydrogels from decellularized tissues have great potential to enhance endothelialization of vascular grafts because they are mimicking the ECM composition of tissues and promote stem cell homing. Decellularized non-human animal tissues are at risk of immune rejection due to DNA or cell remnants. However, various approaches of using human ECMs for tissue engineering purposes show promising results for constructive remodeling after stroke and brain injury, ligament reconstruction and wound healing [40,41]. The human placenta, especially the amniotic membrane, has gained interest due to its favorable characteristics. Placenta tissue is non-immunogenic [42,43] and amnion has already been used as wound dressings in different clinical applications [[43], [44], [45]]. HuBiogel™, a commercially available ECM hydrogel derived from human amnion, was already used for vascular graft coating and showed satisfying endothelialization in-vitro [46]. However, it was not assessed whether HuBiogel™ is comparable or even superior to other coating materials in terms of endothelialization.

Due to the promising results of using amnion for tissue engineering purposes, interest in other parts of the placenta has risen. It was shown before that most of the ECM proteins are very similar in amniotic and chorionic tissue [47]. However, nidogen, matrix metalloproteinases (MMP)-1, MMP-2, MMP-9 and metallopeptidase inhibitor 1 (TIMP-1) can be only found in amnion whereas MMP-9 is present in both tissues [47,48]. Despite these previously reported similarities in composition, Lakkireddy et al. (2022) were able to show that decellularized human chorionic membrane was more efficient to induce adhesion and proliferation than decellularized amnion [49]. Francis et al. (2017) showed that a hydrogel from human chorion reduced scarring after cardiac ischemia in animal studies and depicted beneficial effects on cardiomyocytes, endothelial cells and stem cells in-vitro [50]. Several other studies indicated that decellularized chorion can be used for tissue regeneration purposes [51] or even for the production of vascular grafts when the chorionic membrane is rolled to tubes [52]. Previous studies from our group also documented the usability of decellularized chorionic arteries for small diameter vascular grafting [14,53,54]. The conduits depicted good biocompatibility, low immunogenicity and low thrombogenicity in a rodent infrarenal aortic replacement model [54]. We therefore developed a protocol for the production of a self-made chorionic hydrogel and adapted it for the use as coating material. Chorionic tissue contains considerable amounts of basal lamina proteins fibronectin and laminin as well as collagen subtypes with different composition than amnion. Since contents of the basal lamina mediate endothelial cell adhesion and function [55], we hypothesized that our human placental chorionic ECM-gel (hpcECM-gel) might be a promising candidate for enhancing the endothelialization of ePTFE vascular grafts when used as a coating. We developed a decellularization procedure for chorionic plates which ensured the preservation of ECM proteins and enabled the use of the hpcECM-gel as coating material without containing extensive amounts of growth factors which may influence endothelial cell function in the long-term.

So far, ECM-gel from chorion has not been used for vascular graft coating. The aim of the study was to evaluate if this self-made hpcECM-gel could serve as a coating for ePTFE vascular grafts to induce endothelialization. Fibronectin was used as a positive control in this study. The hpcECM-gel was characterized in terms of DNA removal, preservation of ECM proteins and structural features. Coating efficiency as well as the viability, proliferation, attachment and gene expression of endothelial cells were investigated. Furthermore, materials were tested for hemocompatibility and flow retention in-vitro. Based on these evaluations the potential of the hpcECM-gel as a graft coating material alternative to fibronectin for future clinical applications should be assessed.

## 2 Materials and methods

### 2.1 hpcECM-gel production

Placentas derived from caesarean section births at term (week 37/0–40/0) were obtained from the Department of Obstetrics and Gynaecology at the Medical University of Vienna after written informed consent of the mothers and approval by the institutional ethics committee (EK:1602/2018). The chorion was carefully scratched off the basal tissue after the amnion and umbilical cord were removed. The material was frozen at −80 ​°C for at least 24 ​h prior to use.

Pooled chorionic material from 8 placentas was decellularized with 2% Triton X-100 (Sigma-Aldrich, Austria) and 0.02% ethylenediaminetetraacetic acid (EDTA, Sigma-Aldrich, Austria) for 3 subsequent days each with a 1× concentrated phosphate buffer saline (1xPBS) washing step in between. To remove remaining DNA, the tissue was subjected to an overnight 0.04% DNase (Roche, Switzerland) treatment after intensive 1xPBS washing. The decellularized tissue was sterilized with 0.18% peracetic acid in 4.8% ethanol (both Sigma-Aldrich, Austria) for 2.5 ​h, frozen at −20 ​°C, lyophilized and cryomilled with a FreezerMill 6770 (SpexSamplePrep, USA). All subsequent steps were performed under a sterile laminar flow hood. The chorionic powder was solubilized by enzymatic digestion under acidic conditions (HCl – 0.1 ​M; Merck, Germany) using porcine gastric mucosa pepsin (Roche, Switzerland). Digestion was conducted for 48 ​h under constant stirring at room temperature before neutralizing the gel (NaOH - 1 ​M; Merck, Germany) and adjusting the salt concentration to 1× using 10xPBS. The resulting hpcECM-gel was stored at 8 ​°C before further use.

### 2.2 hpcECM-gel characterization

Specimens from decellularized chorionic tissue were examined by histological staining to confirm the removal of cells post-decellularization as described before [54]. Briefly, tissue pieces (pre-and post-decellularization) were fixed in 4% formaldehyde solution for 24 ​h at 4 ​°C. Thereafter, they were washed with tap water and transferred to 50% ethanol solution for 1 ​h and further stored in 70% ethanol. Dehydration was completed with an ascending series of alcohol. Then the specimens were embedded in paraffin wax, sectioned and placed on slides. Sections were stained for hematoxylin and eosin (H&E) according to the manufacturer's protocol (Morphisto, Germany).

Further investigations were carried out directly on the pre-gel solutions. Pre-gel solutions produced from blood free native tissues were used as control. Biochemical quantification methods to determine residual DNA, elastin, glycosaminoglycan (GAG) and collagen content in hpcECM pre-gels were performed as described in our previous study [53]. Briefly, DNA was extracted via a Tissue DNA Extraction Mini Kit (Favorprep™, Taiwan) and quantified using an AccuBlue® High Sensitivity dsDNA Quantitation Kit (Biotium, USA). Collagen content in pre-gel samples was determined by measuring the hydroxyproline content of neutralized samples after hydrolysis in 6 ​M HCl at 95 ​°C for 20 ​h. To directly measure the sulphated glycosaminoglycan (GAG) content, dilutions of pre-gel samples were stained with 1,9-dimethyl- methylene blue (DMB, Sigma-Aldrich, Austria) and photometrically measured at 525 ​nm. To investigate the amount of elastin, a Fastin elastin assay (Biocolor, UK) was conducted according to the manufacturer's protocol. Sandwich enzyme-linked immunosorbent assays (ELISAs) were performed to determine the amount of fibronectin and laminin in the hpcECM-gel by using ELISA kits from Cohesion Bioscience (United Kingdom) according to the manufacturer's instructions. Briefly, three different hpcECM-gel hydrogel batches were diluted to 10 ​mg/mL and subjected to the ELISA plates. Intense washing steps were performed and detection antibody cocktails as well as horseradish peroxidase (HRP)-linked streptavidin were added. A detectable signal was generated by administrating 3,3′, 5,5″- tetramethylbenzidine and absorbance was measured with a Thermo Varioskan Flash device (Thermo Scientific, USA).

### 2.3 Structural analysis of hpcECM-gel

Scanning electron microscopy was used to visualize the fibrous ultrastructure of the hpcECM-gel after gelation. Therefore, 1 ​mL of 0.2 ​mg/mL hpcECM pre-gel solution was pipetted onto a circular cover glass and incubated at 37 ​°C to facilitate gelation, then fixed in 2.5% glutaraldehyde overnight at 4 ​°C and dehydrated with an increasing ethanol series with a final hexamethyldisilazane (HMDS) step. Chemically dried samples were sputter coated with 20 ​nm of gold and analyzed with a Zeiss EVO 10 (Zeiss, Austria).

### 2.4 ePTFE graft coating

Aeos™ ePTFE grafts (Extruded Sub-Lite-Wall®, inner diameter 0.06’’ ±0.002″, wall thickness 0.004’’ ±0.001″) were purchased from Zeus Industrial Products (USA). Grafts were either coated with 50 ​μg/mL human fibronectin (Sigma-Aldrich, Austria), 200 ​μg/mL hpcECM-gel or placed in 1xPBS (uncoated condition) for 30 ​min in a common cell culture incubator at 37 ​°C. Grafts were then washed twice with 1xPBS and used for experiments. All conditions will be referred to as “coated grafts”.

### 2.5 Coating visualization

For scanning electron microscopy imaging, coated ePTFE grafts (50 ​μg/mL fibronectin, 200 ​μg/mL hpcECM-gel) were fixed with 2.5% glutaraldehyde overnight at 4 ​°C and further subjected to an increasing ethanol series (25% up to 100%). As a final step, samples were dried in hexamethyldisilazane (HMDS, Sigma-Aldrich, Austria) overnight. Graft pieces were then sputter coated with 20 ​nm gold and imaged with a Zeiss EVO 10 (Zeiss, Austria).

Coating was further visualized by immunofluorescence staining. Coated grafts were fixed with 4% formaldehyde overnight at 4 ​°C, triple washed with 1xPBS supplemented with 1% bovine serum albumin (PBS/1% BSA, Sigma-Aldrich, Austria) and incubated with primary antibodies mouse anti-human fibronectin (Abcam, USA) and mouse anti-human collagen (Abcam, USA) for 1 ​h at room temperature and then subjected to secondary antibody goat anti-mouse Alexa 647 antibody (Life Technologies, USA) for another hour. Samples were placed on microscopy slides with fluorescence mounting medium (Agilent, USA) supplemented with 1 ​μM 4’,6-Diamidin-2-phenylindol (DAPI, Sigma-Aldrich, Austria) and imaged with a LSM 700 confocal microscope (Zeiss, Germany).

### 2.6 Cell culture

Pooled donor human umbilical vein endothelial cells (HUVECs) were purchased from Lonza (Switzerland) and cultivated in Endothelial Growth Medium-2 (EGM-2, Lonza, Switzerland) supplemented with additional 10% fetal bovine serum (FBS, Sigma-Aldrich, Austria) and 1% penicillin/streptomycin (Gibco, USA). Medium change was performed every second day and cells were passaged at 80% confluence. HUVECs were used between passages 3 and 8.

### 2.7 Cell biocompatibility assays

In order to assess the amount of living and dead cells on coated ePTFE grafts, 9 ​mm^2^ graft pieces were subjected to 96 well cell crowns (Sigma-Aldrich, Austria) and seeded with 5 ​× ​10^4^ HUVECs per graft piece for 10 ​min. Unattached cells were washed away and the specimens were incubated for 24 ​h under standard cell culture conditions. Cells were stained with 2.5 ​μg/mL propidium iodide (dead cells, Sigma-Aldrich, Austria) and 1 ​μg/mL Calcein AM (live cells, Biotium, USA) for 30 ​min in full EGM-2 at 37 ​°C. Cells were triple washed with 1xPBS+/+ each for 1 ​min and then fixed with 4% formaldehyde (Sigma-Aldrich, Austria) overnight at 4 ​°C. Graft pieces were mounted to microscopy slides with fluorescence mounting medium supplemented with 1 ​μM DAPI and imaged with a LSM 700 confocal microscope.

Furthermore, another viability assay was performed. HUVECs were seeded to coated ePTFE grafts (4 ​× ​6 mm pieces, 5 ​× ​10^4^ ​cells per patch) and cultured for up to 72 ​h under standard cell culture conditions. 2,3-Bis(2-methoxy-4-nitro-5-sulfophenyl)-2H-tetrazolium-5-carboxanilide inner salt (XTT, Santa Cruz Biotechnology, USA) was dissolved in EGM-2 for 20 ​min at 60 ​°C on a thermomixer. 0.25 ​mM phenazine methosulfate (PMS, Sigma-Aldrich, Austria) was added and cell-seeded samples were incubated in a 1:10 dilution with EGM-2 and the prepared XTT/PMS working solution for 3 ​h at 37 ​°C. Subsequently, the cell culture supernatant was transferred to a new 96-well plate and absorbance was measured at 450 ​nm (620 ​nm reference wavelength) with a SPARK® Multimode microplate reader (TECAN, Austria).

### 2.8 Cell proliferation

To determine the proliferation rate of HUVECs on coated ePTFE grafts, cells were seeded to 24-well plates (Corning, USA) in a concentration of 5 ​× ​10^3^ ​cells/well. Every 24 ​h for three days, cells were detached with trypsin (Gibco, USA) and counted manually with a Neubauer counting chamber (Sigma-Aldrich, Austria).

### 2.9 Cell attachment assay

HUVECs were seeded to coated ePTFE patches on cell crowns for 10 ​min (5 ​× ​10^4^ ​cells/graft) at 37 ​°C. Subsequently, grafts were carefully triple washed with 1xPBS to remove unattached cells. Adhered HUVECs were fixed with 4% formaldehyde overnight at 4 ​°C and stained with DAPI the next day. Images were taken with an inverse fluorescence microscope (Zeiss, Germany) and cell numbers were determined with the ImageJ cell counter plugin (ImageJ, NIH, USA).

### 2.10 Endothelial progenitor cell (EPC) isolation

Healthy male and female volunteers without anti-coagulant therapy were subjected to venepuncture after approval of the local ethics committee (EK: 2321/2020) and 45 ​mL of blood was collected into 3.2% sodium citrate tubes (Vacuette, Greiner, Bio-One, Austria). The peripheral blood mononuclear cell (PBMC) fraction was isolated and washed twice with 1xPBS centrifugation steps. Cells were subsequently resuspended with 10 ​mL of EGM-2. Grafts were coated with 50 ​μg/mL fibronectin, 200 ​μg/mL hpcECM-gel or were left uncoated as described above. Cells were directly seeded to the graft specimens after isolation (3 ​× ​10^6^ ​cells per graft in EGM-2) for 24 ​h before a first medium change was performed. Grafts were then cultured for 1 week under standard cell culture conditions with medium changes every 2–3 days. Grafts were washed with 1xPBS twice, fixed with 4% formaldehyde overnight, then stained with mouse anti-human Prominin-1 antibody (Prom-1, Invitrogen, USA), further with secondary goat anti-mouse Alexa 647 antibody (Life Technologies, USA) and imaged with a Zeiss LSM700 confocal microscope (Zeiss, Germany).

### 2.11 Integrin western blot

HUVECs were either seeded for 10 ​min or 72 ​h to non-coated, fibronectin- or hpcECM-gel-coated polystyrene petri dishes (150 ​mm, 3 ​× ​10^6^ ​cells per dish). Cells were lysed in 100 ​μL lysis buffer (20 ​mM Trizma base, 0.137 ​M NaCl, 10% glycerine and 10 ​mM EDTA in dH_2_O, all acquired from Sigma-Aldrich, Austria) and incubated on ice for 30 ​min. After 40 ​min centrifugation with 14,000 ​g, samples were stored at −80 ​°C until further use. Protein quantification was done using a Pierce™ Modified Lowry Protein Assay Kit (Thermo Scientific, USA) and samples were adjusted to 20 μg/10 ​μL with 6× Laemmli buffer (0.375 ​M Tris pH 6.8, 12% SDS, 60% glycerol, 0.6 ​M DTT, 0.06% bromophenol blue), heated up to 95 ​°C for 10 ​min and frozen at −20 ​°C until gel loading. Samples were loaded to 10% SDS-gels and run at 40 ​V per gel for 1.5 ​h. Subsequently, proteins were blotted to polyvinylidene difluoride (PVDF) membranes (pre-conditioned with methanol and dH_2_O) in a tank blotting system (Bio-Rad, Austria) at 320 ​mA for 30 ​min. After a 1 ​h blocking period with 5% milk in Tris buffered saline with 0.1% Tween20 (TBST), membranes were incubated with primary rabbit anti-human antibodies anti-α5-, anti-β1- and anti-β3 antibodies (1:1000 dilution, Integrin Antibody Sampler Kit, CST, USA) overnight at 4 ​°C on a roller shaker. The next day, membranes were triple washed with TBST followed by 45 ​min incubation with goat-*anti*-rabbit HRP-linked antibody and a subsequent washing step with TBST (three times). Finally, visualization was done via a homemade ECL solution (0.1 ​M Tris pH ​= ​8.6, 13.07 ​mg/mL Coumaric acid, 0.044 ​mg/mL Luminol, 3% H_2_O_2_) using a Chemidoc imaging system (Bio-Rad, Austria). After detection, membranes were blocked again and further incubated with HRP-linked mouse anti-human β-actin (clone AC-15, 1:5000 dilution, Abcam) and again imaged as described above. Quantification was performed with ImageJ (NIH, USA).

### 2.12 Endothelial cell activation assessment

HUVECs which were cultured on coated grafts were triple washed with 1xPBS and fixed in 4% formaldehyde overnight at 4 ​°C. Subsequently, samples were washed with PBS/1% BSA three times to remove remaining formaldehyde. Primary antibody staining with fluorescein isothiocyanate (FITC) labelled mouse anti-human platelet endothelial cellular adhesion molecule-1 (1:50, PECAM-1, BD Pharmingen, USA), Alexa Fluor 555 labelled phalloidin (1:1000, Abcam, UK), unlabelled mouse anti-human vascular cellular adhesion molecule-1 (1:200, VCAM-1, Santa Cruz Biotechnology, USA) or unlabelled mouse anti-human intercellular adhesion molecule-1 (1:200, ICAM-1, Santa Cruz Biotechnology, USA) was performed for 1 ​h at room temperature in humid environment. Specimens were triple washed with PBS/1% BSA. Unlabelled VCAM-1 and ICAM-1 were further stained with secondary goat anti-mouse Alexa Fluor 647 labelled antibody (1:200, Life Technologies, USA) for 1 ​h at room temperature. The grafts were subjected to a final washing step with PBS/1% BSA and mounted to microscopy slides with fluorescent mounting medium with 1 ​μM DAPI as described above. All immunofluorescence staining was imaged with a Zeiss LSM 700 confocal microscope.

Quantification of fluorescence intensity was performed with ImageJ. Images were changed to 8-bit format first. Cells were then selected with a free-hand selection tool and integrated density, area as well as mean grey value were measured. The corrected total cell fluorescence (CTCF) was calculated as follows: CTCF = Integrated density – (area x mean of background) for each cell.

The gene expression of ICAM-1, VCAM-1 and PECAM-1 was determined by reverse transcription quantitative polymerase chain reaction (RT-qPCR). RNA was isolated from HUVECs cultured on coated ePTFE grafts for 72 ​h under static condition with a RNeasy mini kit (Qiagen, Germany) according to the manufacturer's manual. RNA amount and quality was determined with a SPARK microplate reader and further reversely transcribed to cDNA with a reverse transcription kit (Qiagen, Germany) again according to manufacturer's instructions. 30 ​ng cDNA per sample were pipetted to SYBR Green PCR master mix (Applied Biosystems, USA) and 10 ​μM of the respective primers (See Table 1) were added. RT-qPCR was performed on a realplex thermocycler (Eppendorf, Germany) with standard enzyme and cycling conditions.

**Table 1:** Transcript specific primers for RT-qPCR.

Name	Forward primer	Reverse Primer
GAPDH	TGGGAAGCTGGTCATCAAC	GCATCACCCCATTTGATGTT
ICAM-1	ATGCCCAGACATCTGTGTCC	GGGGTCTCTATGCCCAACAA
VCAM-1	GGGAAGATGGTCGTGATCCTT	TCTGGGGTGGTCTCGATTTTA
PECAM-1	GCAACACAGTCCAGATAGTCG	GACCTCAAACTGGGCATCAT

### 2.13 HUVEC retention to flow

Coated ePTFE grafts were subjected to a simple perfusion set-up consisting of a Reglo ICC rollerpump (Ismatec, ICC), silicone tubes (2 ​mm inner diameter, Saint Gobain, France), a self-poured silicone (SYLGARD™, Dow, USA) chamber with needles (Neoject 16G, Dispomed, Germany) to fix the grafts with 4/0 polypropylene suture (Prolene, Ethicon, Germany). 3 ​× ​10^6^ HUVECs per graft were seeded for 3 ​h to allow preliminary cell attachment. Grafts were reseeded under low perfusion conditions with 50 ​μL/min flow for 72 ​h. Fixation and staining with phalloidin and DAPI was performed as described above.

To determine cell retention under physiological flow conditions, cell seeded grafts were subjected to a home-made bioreactor system for 6 ​h at a perfusion rate of 20 ​mL/min 3 ​× ​10^6^ HUVECs were seeded per graft for 24 ​h before perfusion to ensure a completed cell attachment process. Remaining cells after perfusion were determined by detachment and manual counting.

### 2.14 Hemocompatibility assays

Blood clotting assay was performed by coating ePTFE patches (4 ​× ​4 mm) as described above. After coating, graft pieces were weighed. Freshly donated human blood from healthy volunteers without anticoagulant therapy was collected by venepuncture into 3.2% sodium citrate tubes (Vacuette, Greiner Bio-One, Austria) and then re-calcified with a final concentration of 10 ​mM CaCl_2_ (Sigma-Aldrich, Austria). Round glass coverslips with a diameter of 10 ​mm (VWR International, Austria) were used as positive controls. The graft pieces were incubated in the re-calcified blood for 1 ​h at 37 ​°C and weighed again. Elevated mass was considered as an indication of thrombus formation on the surface.

To assess the hemolytic potential of hpcECM-gel, blood collection and graft preparation was performed as described above for blood clotting assays. The blood was diluted in a 1:5 dilution with 0.9% NaCl and coated graft pieces were incubated in the dilution for 1 ​h at 37 ​°C. A 1:5 dilution of blood with distilled H_2_O served as a positive control, whereas a 1:5 blood to 0.9% NaCl dilution without any ePTFE specimen was used as a negative control. After the incubation time, samples were centrifuged for 5 ​min at 3000 ​rpm and absorbance of the supernatants was measured with a Spark multiplate reader at 541 ​nm wavelength. The hemolysis rate was calculated according to the following formula:

((*Absorbance[samp]*-*Absorbance[neg]*)/(*Absorbance[pos]*-*Absorbance[neg]*))∗100%

Platelet adhesion was determined by blood collection as described above, diluted 1:1 with Ficoll® Paque Plus (Sigma-Aldrich, Austria) and centrifuged for 30 ​min at 300×*g* without brake to separate cell layers. The upper layer was transferred to a new falcon tube and filled up to 50 ​mL with PBS. The suspension was centrifuged for 10 ​min at 300×*g* and the upper 2/3 of the solution (platelet-rich plasma) was seeded to coated grafts for 2 ​h at 37 ​°C. Grafts were then fixed with 2.5% glutaraldehyde overnight at 4 ​°C and further dehydrated as described for SEM above. After dehydration, the graft pieces were stained with 10 ​mM mepacrine (Sigma-Aldrich, Austria) in dH_2_O for 1 ​h at room temperature in the dark. Grafts were washed with 1xPBS until no yellow colour was observed on the surface of the grafts. Imaging was performed with a LSM700 confocal microscope (Zeiss, Germany).

### 2.15 Statistics

All experiments were planned as balanced designs. One-way ANOVA was conducted in case of equal variance presence and Kruskal-Wallis test for non-parametric testing. To compare the means of all groups during multiple comparison analyses, Tukey's post-hoc test was performed. *p* ​< ​0.05 was regarded as significant. All graphs are displayed as mean ​± ​standard deviation.

## 3 Results

### 3.1 hpcECM-gel characterization

**Fig. 1:**
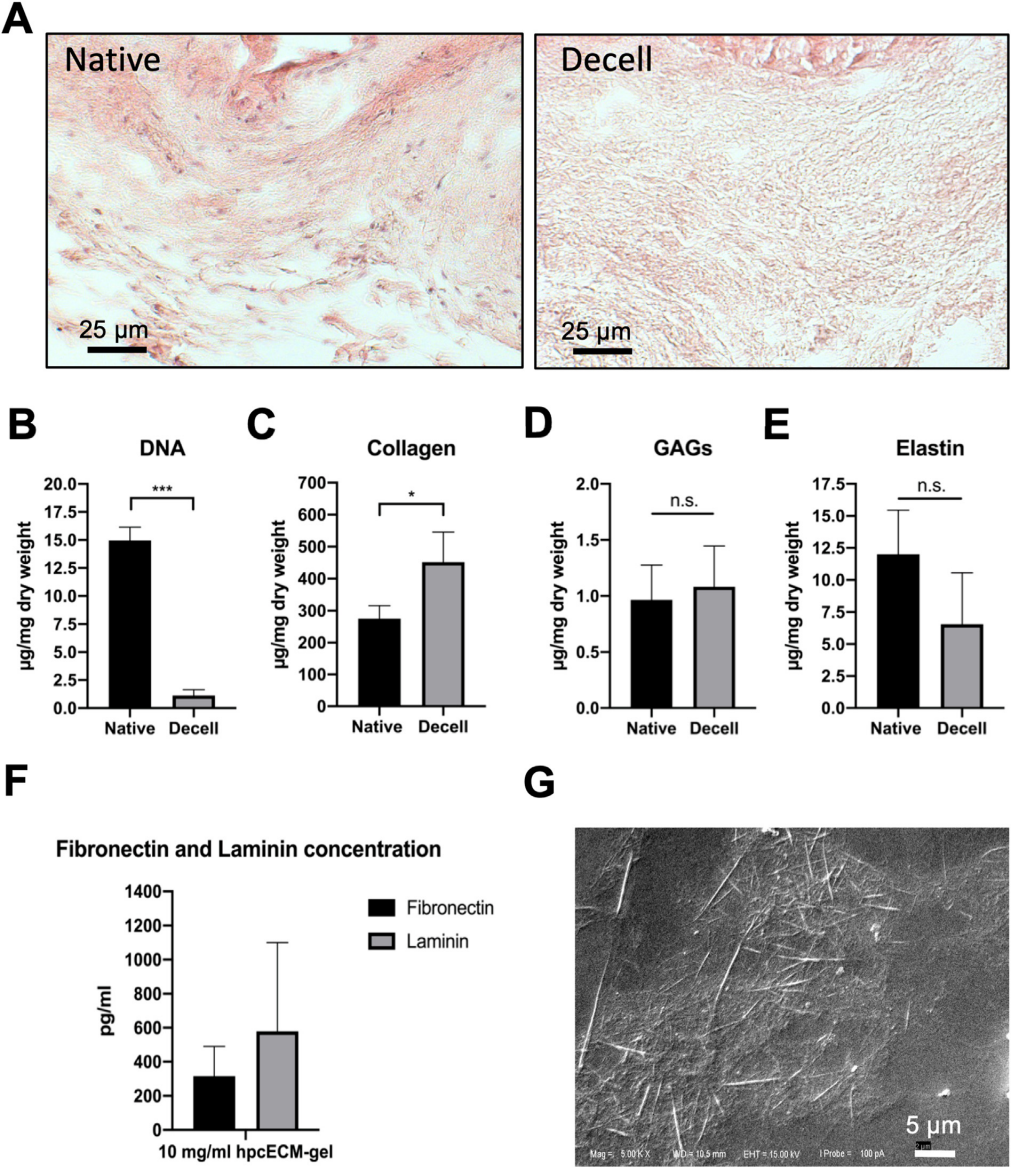
Hydrogel characterization. Chorionic tissue was successfully decellularized with no visible cells remaining after the decellularization process (A). The amount of DNA was significantly reduced after the decellularization process (B). Collagen (C), GAGs (D) and Elastin (E) were preserved after decellularization. 10 ​mg/mL hpcECM-gels showed fibronectin and laminin content (F). The hydrogel structure depicts ECM fibres distributed over the coated glass surface by scanning electron microscopic imaging (magnification ×10000) (G).

Prior to the use of hpcECM-gel, the decellularization efficiency was assessed. H&E staining revealed complete removal of cells in the resulting tissue matrix after decellularization (Fig. 1A). DNA quantification confirmed a significant reduction of DNA content in decellularized tissue compared to controls (Native: 13.61 ​± ​3.18 ​μg, Decell: 1.13 ​± ​0.6 ​μg DNA/mg dry weight) (Fig. 1B). Since enzymatic tissue decellularization might lead to a remarkable loss or degradation of important ECM-proteins, the post-decellularization content of ECM-proteins was quantified. Hydroxyproline assay revealed substantial preservation of collagen (Native: 228.4 ​μg, Decell: 451.8 ​μg collagen/mg dry weight) (Fig. 1C). In addition, the content of GAGs (Native: 1.016 ​μg, Decell: 1.08 ​± ​0.41 ​μg GAGs/mg dry weight) (Fig. 1D) as well as elastin (Native: 12.28 ​μg, Decell: 6.56 ​± ​4.63 ​μg elastin/mg dry weight) was reduced after decellularization (Fig. 1E). To evaluate if ECM-proteins important for endothelial cell adhesion were present in the hpcECM-gel, ELISAs were performed to determine the amounts of fibronectin and laminin. Fig. 1F demonstrates that fibronectin and laminin were detected with batch-related differences in 10 ​mg/mL hpcECM-gels (Fibronectin: 315.10 ​± ​175.44 ​pg/mL, Laminin: 578.69 ​± ​521.53 ​pg/mL).

### 3.2 hpcECM-gel structure and visualization

**Fig. 2:**
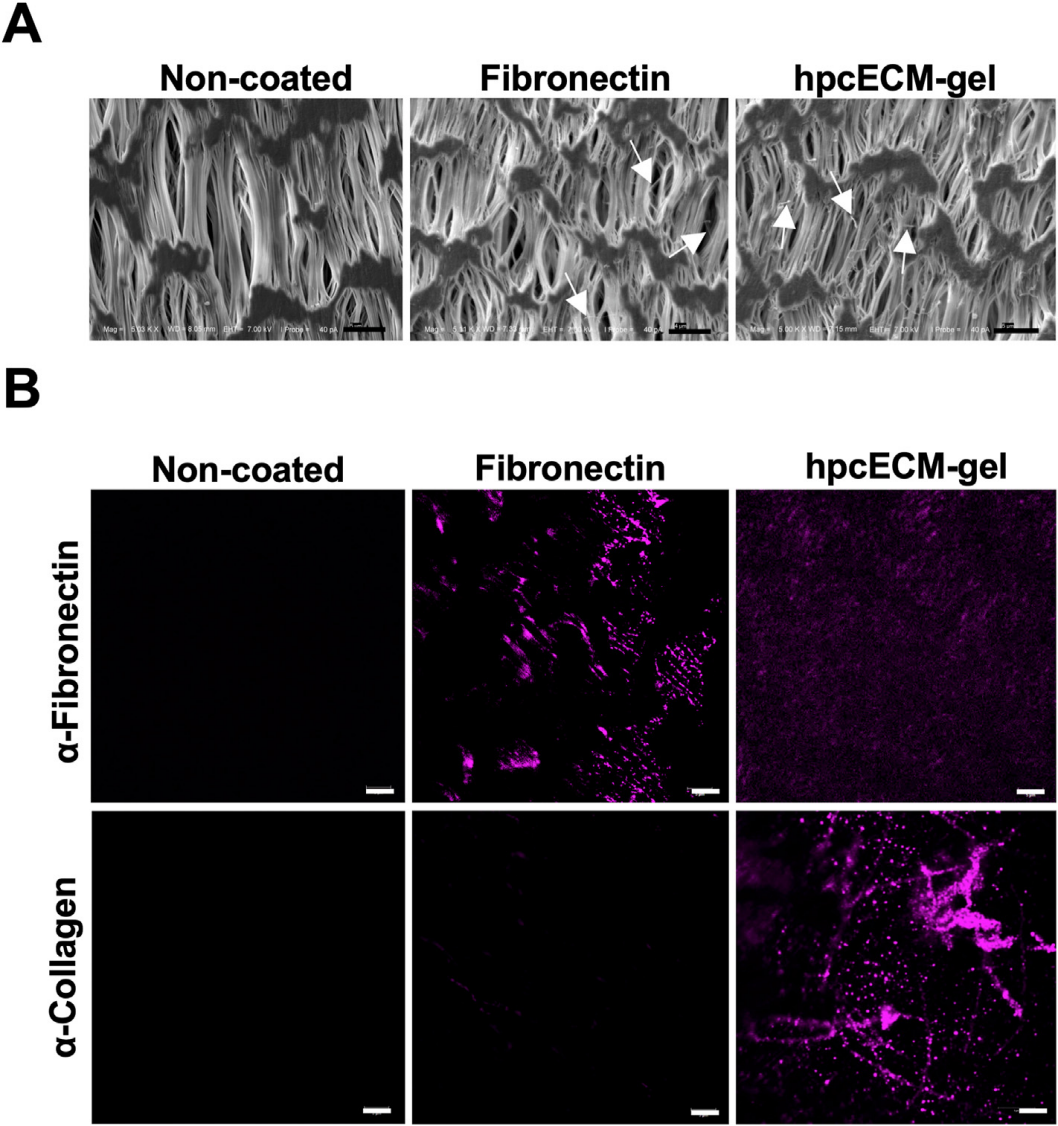
Evaluation of the coating efficiency of ePTFE grafts: non-coated, fibronectin coated (50 ​μg/mL) and hpcECM-gel coated (200 ​μg/mL) specimens. SEM images depict thin fibers on the coated graft surfaces (Scale bar ​= ​5 ​μm) ​(A). Immunofluorescence images showed fibronectin positive staining on the fibronectin-coated grafts. HpcECM-gel coated grafts revealed collagen positive fibers and a faint signal for fibronectin (Scale bar ​= ​5 ​μm) ​(B).

To ascertain that the hpcECM-gel coating is present on the coated surfaces, visualization strategies were applied. SEM revealed a loose collagen fibrous structure of hpcECM-gel at a concentration of 0.2 ​mg/mL on glass surfaces (Fig. 1F). SEM visualization in Fig. 2A demonstrates the presence of fibronectin and hpcECM-gel on cell-free ePTFE grafts after the standard coating procedure for 30 ​min incubation. Thin filaments of coating materials were spread over the surface (indicated by arrows). The specimens were subjected to immunofluorescence staining for further visualization. Non-coated samples served as negative controls and did not show any positive signals for fibronectin or collagen. Fibronectin-coated samples demonstrated a clear signal for the presence of fibronectin. It was further shown that hpcECM-gel coating resulted in collagen I positive signal on the hpcECM-gel coated graft surface, whereas for fibronectin, no significant staining for collagen was detected. Interestingly, hpcECM-gel coated samples demonstrated a faint signal for fibronectin after staining (Fig. 2B).

### 3.3 hpcECM-gel depicts endothelial cell biocompatibility

To exclude possible cytotoxic effects of hpcECM-gel, HUVECs were seeded on coated tissue culture plates in preliminary experiments. HUVECs seeded on either non-coated or hpcECM-gel coated surfaces showed unimpaired viability both, in a Live/Dead staining assay and during XTT measurement (Non-coated: 1.06 ​± ​0.34 AU, Fibronectin: 1.14 ​± ​0.38 AU, hpcECM-gel: 1.24 ​± ​0.44 AU after 96 ​h, Suppl.Fig. A&B). The migration behavior of HUVECs was not changed on hpcECM-gel compared to the other conditions in a simple scratch assay (Fold reduction of scratched area: Non-coated: 1.23 ​± ​0.12, Fibronectin: 1.20 ​± ​0.11, hpcECM-gel: 1.21 ​± ​0.13) (Suppl.Fig. C). However, the initial adhesion was significantly upregulated on hpcECM-gel (Uncoated: 109 ​± ​121 ​cells, hpcECM-gel: 320 ​± ​218, Suppl.Fig. D). Further experiments were all performed on hpcECM-gel coated ePTFE grafts.

### 3.4 HpcECM-gel significantly enhances endothelial cell attachment on ePTFE grafts

**Fig. 3:**
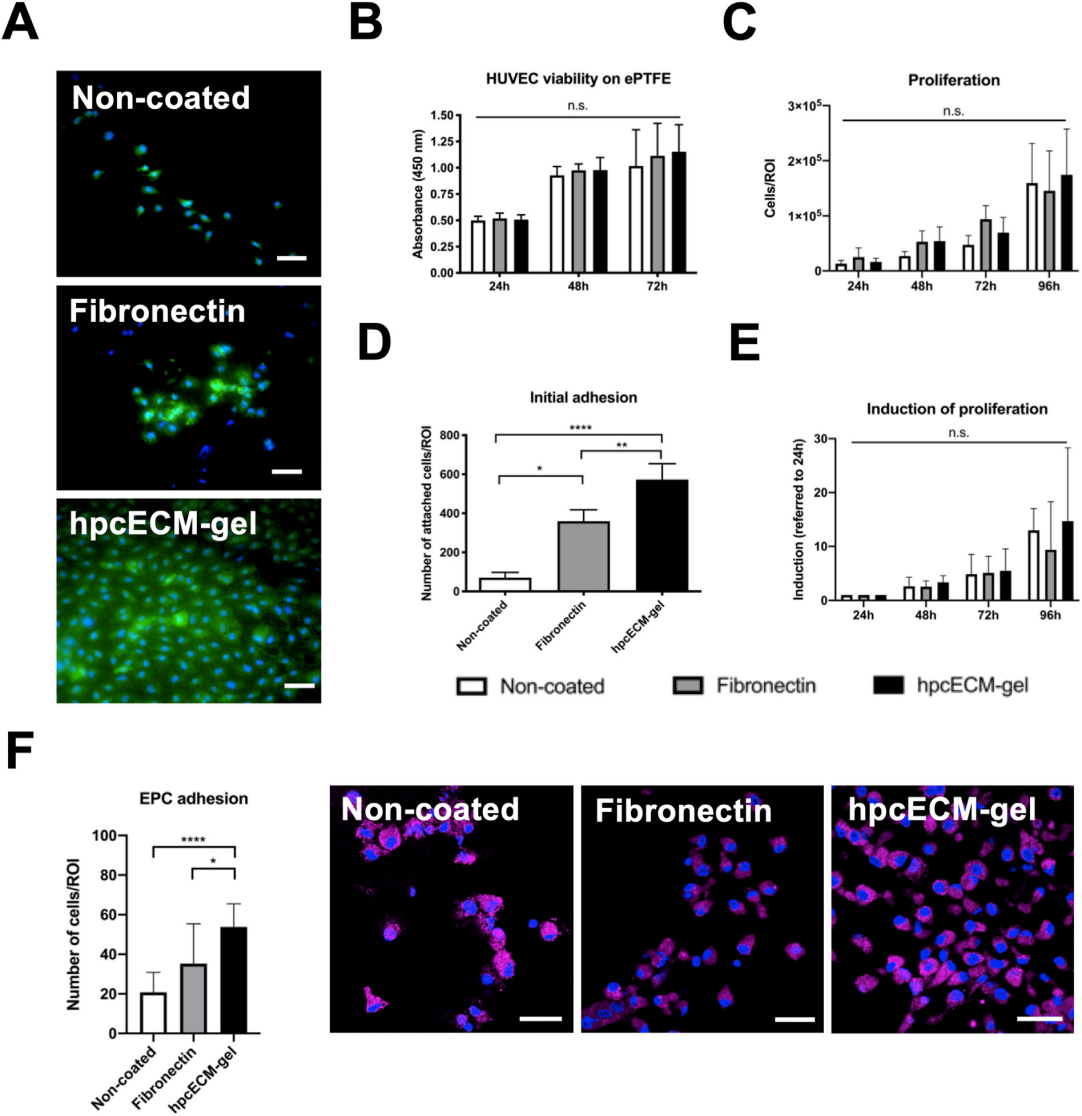
Cytocompatibility, proliferation and adhesion of endothelial cells on coated ePTFE grafts. An increased number of viable cells was observed on hpcECM-gel coated grafts (Calcein AM/Propidium Iodide staining) after 24 ​h (A), but no significant changes in cell viability (B) or proliferation rates (C) were observed over a time period up to 96 ​h. HUVEC adhesion was significantly upregulated in cells seeded on hpcECM-gel compared to fibronectin or non-coated surfaces after 10 ​min of seeding time (D). Proliferation rates of cells which showed higher attachment in the adhesion assay however do not proliferate faster on hpcECM-gel compared to the other conditions (E). An increased number of EPCs were found on hpcECM-gel coated ePTFE grafts after one week of incubation (Prom-1 staining) (F). Scale bars ​= ​50 ​μm, n ​= ​6.

Experiments revealed an efficient decellularization with substantial ECM protein preservation, the presence of collagen fibers after the coating period and significantly increased HUVEC adhesion on hpcECM-gel compared to non-coated and fibronectin-coated tissue culture plates. Cell viability of HUVECs was determined by Live/Dead staining and a XTT cell viability assay. Fig. 3A demonstrates that the number of viable cells (stained with Calcein AM) was not compromised neither by fibronectin nor by hpcECM-gel coating. The typical cobblestone-like morphology of HUVECs was most prominent on hpcECM-gel coated grafts. Interestingly, the staining further showed that considerably more cells attached on the hpcECM-gel coated ePTFE graft after 24 ​h. For this experiment, cells were seeded for 10 ​min and non-attached cells were removed after this short seeding time. The attached cells were then further incubated for 24 ​h. Since the performed XTT assay (Non-coated: 1.16 ​± ​0.22, Fibronectin: 1.09 ​± ​0.32, hpcECM-gel: 1.13 ​± ​0.27) (Fig. 3B) and further proliferation curves (Non-coated: 1.6 ​× ​10^5^ ​± ​0.7 ​× ​10^5^, Fibronectin: 1.5 ​× ​10^5^ ​± ​0.73 ​× ​10^5^, hpcECM-gel: 1.7 ​× ​10^5^ ​± ​0.8 ​× ​10^5^ ​cells) (Fig. 3C) did not implicate a higher proliferation rate of HUVECs on hpcECM-gel, the adhesion of cells on coated ePTFE grafts was determined. The initial adhesion of cells was significantly enhanced on hpcECM-gel (573 ​± ​92 ​cells) compared to fibronectin (360 ​± ​73 ​cells) or non-coated grafts (70 ​± ​28 ​cells) (Fig. 3D) during an attachment assay. The absence of changes seen in proliferation might be due to the fact that all cells seeded to the wells were analyzed and not exclusively HUVECs that attached after 10 ​min. Subsequently, only short-term adhered cells (10 ​min) were observed for proliferation. Interestingly, there was a trend towards increased induction of proliferation on non-coated surfaces after 96 ​h compared to fibronectin (Non-coated: 12.99 ​± ​4.02, Fibronectin: 9.39 ​± ​8.88, hpcECM-gel: 14.69 ​± ​13.62) (Fig. 3E).

Since fall-out endothelialization from endothelial progenitors happens by attachment of EPCs on the graft surface during in-vivo implantation, EPCs were isolated from freshly donated human blood, seeded on the coated grafts and subjected to EPC-marker Prom-1 immunostaining. The results depicted in Fig. 3F clearly demonstrate a significant increase of EPC adhesion on hpcECM-gel coated grafts (Non-coated: 21 ​± ​10, Fibronectin: 35 ​± ​20, hpcECM-gel: 54 ​± ​12 ​cells).

### 3.5 No significant integrin upregulation on fibronectin and hpcECM-gel seeded cells

**Fig. 4:**
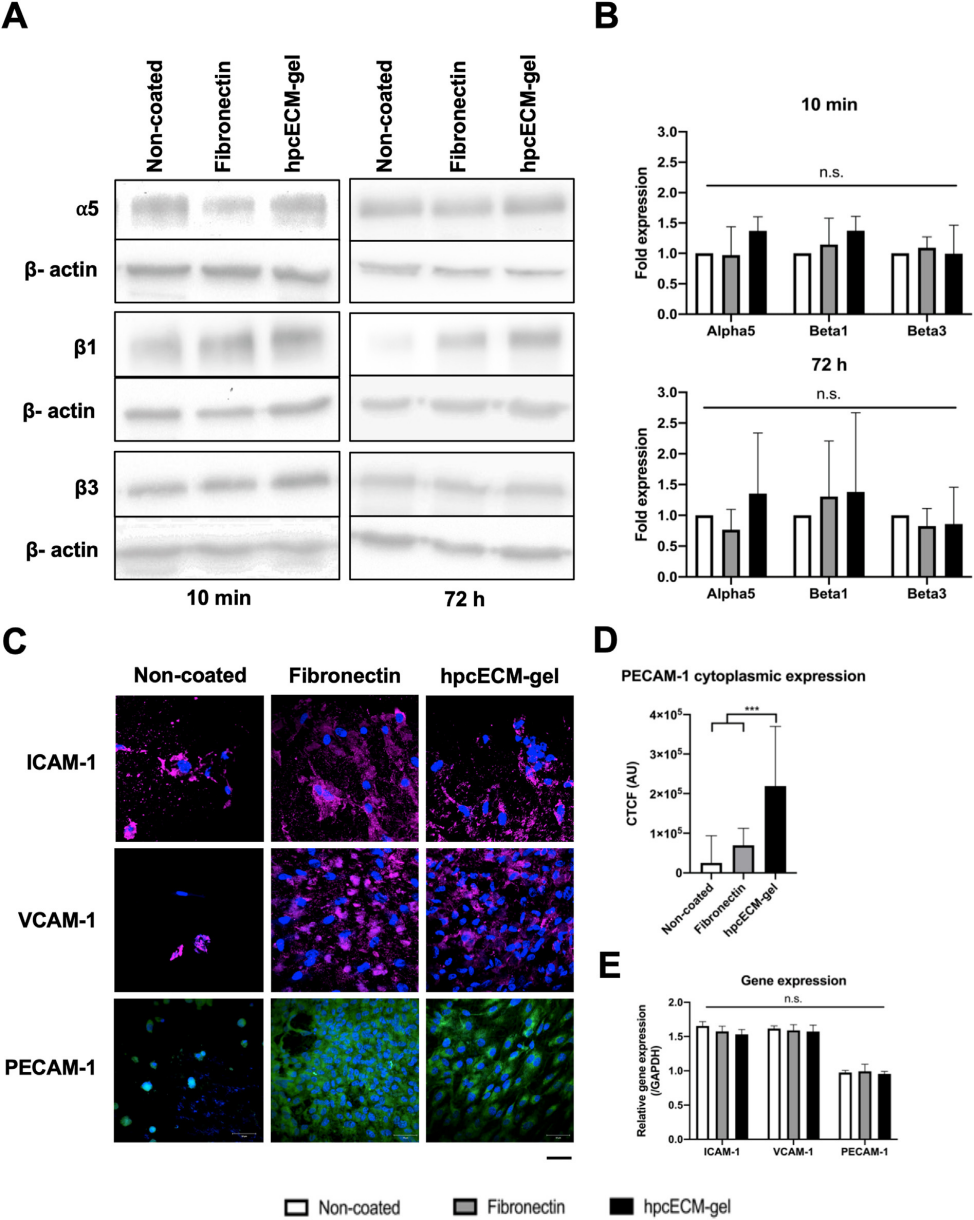
Adhesion molecule expressions. HUVECs seeded on hpcECM-gel coated ePTFE grafts for 72 ​h under static culture conditions show an insignificant increase of alpha5 and beta1 integrin levels (A) and (B) (n ​= ​6). The expression of pro-inflammatory adhesion markers ICAM-1 and VCAM-1 was not induced by hpcECM-gel or by fibronectin. PECAM-1 levels remained unchanged between the conditions (C). Significantly more cytoplasmic PECAM-1 expression was detected on cells seeded on hpcECM-gel (D) (n ​= ​40). RT-qPCR analyses revealed no change in adhesion molecule gene expression after 72 ​h of seeding (E) (n ​= ​3). Scale bar ​= ​50 ​μm.

The previous results depicting higher attachment of HUVECs and EPCs onto hpcECM-gel led to the conclusion that integrins might be upregulated on these cells. Therefore, western blot analyses of the most common integrins found on endothelial cells were performed (Fig. 4A and B). The fold expression of the β3 integrin subunit (normalized to the non-coated condition) remained unchanged after 10 ​min as well as 72 ​h seeding time (Fibronectin: 0.89 ​± ​0.26, hpcECM-gel: 0.99 ​± ​0.47). Furthermore, significantly higher expression of the α5 (Fibronectin: 0.97 ​± ​0.47, hpcECM-gel: 1.37 ​± ​0.23) and β1 monomers (Fibronectin: 1.15 ​± ​0.43, hpcECM-gel: 1.37 ​± ​0.22) were not detected. After 10 ​min of adhesion time, expression values were similar between fibronectin- and hpcECM-gel coated conditions. However, after 72 ​h, the α5 (Fibronectin: 0.7 ​± ​0.32, hpcECM-gel: 1.57 ​± ​0.94) and β1 (Fibronectin: 0.97 ​± ​0.42, hpcECM-gel: 1.54 ​± ​1.37) expression was insignificantly higher on the hpcECM-gel.

### 3.6 HpcECM-gel coating does not lead to endothelial cell activation

To evaluate if the coatings induce unwanted pro-inflammatory changes of HUVEC adhesion marker expression, immunofluorescence staining with antibodies against ICAM-1, VCAM-1 and PECAM-1 were performed after 72 ​h of culturing under static conditions. ICAM-1 and VCAM-1 expression seemed to be enhanced on some cells seeded on non-coated surfaces, whereas individual HUVECs remained without expression of any adhesion molecules (Fig. 4C). ICAM-1 and VCAM-1 expression on HUVECs seeded on fibronectin-coated ePTFE grafts was more equally distributed over the cell surface than in the non-coated condition. HUVECs seeded on hpcECM-gel coated surfaces demonstrated similar ICAM-1 and VCAM-1 expression as cells on fibronectin. PECAM-1 expression was almost absent in HUVECs cultured on non-coated ePTFE, whereas cells seeded on fibronectin or hpcECM-gel coated grafts showed higher PECAM-1 expression. Interestingly, PECAM-1 expression was significantly increased in the peri-nuclear area of HUVECs on hpcECM-gel and more evenly distributed in the cytoplasm of cells seeded on fibronectin (Non-coated: 25124.69 ​± ​68647.72, Fibronectin: 69476.07 ​± ​43011.24, hpcECM-gel: 219026.40 ​± ​151136.09) (Fig. 4D). To determine if there were changes on the gene expression level of these adhesion molecules, a RT-qPCR was performed. No significant differences were observed in expression of all studied genes between the groups (Fig. 4E). However, cells seeded on hpcECM-gel showed a slight reduction of ICAM-1 (Non-coated: 1.66 ​± ​0.06, Fibronectin: 1.58 ​± ​0.08, hpcECM-gel: 1.53 ​± ​0.07) and VCAM-1 (Non-coated: 1.62 ​± ​0.04, Fibronectin: 1.59 ​± ​0.09, hpcECM-gel: 1.57 ​± ​0.09) whereas PECAM-1 expression remained unchanged (Non-coated: 0.97 ​± ​0.03, Fibronectin: 0.99 ​± ​0.1, hpcECM-gel: 0.95 ​± ​0.04).

### 3.7 Endothelial cell retention to flow

**Fig. 5:**
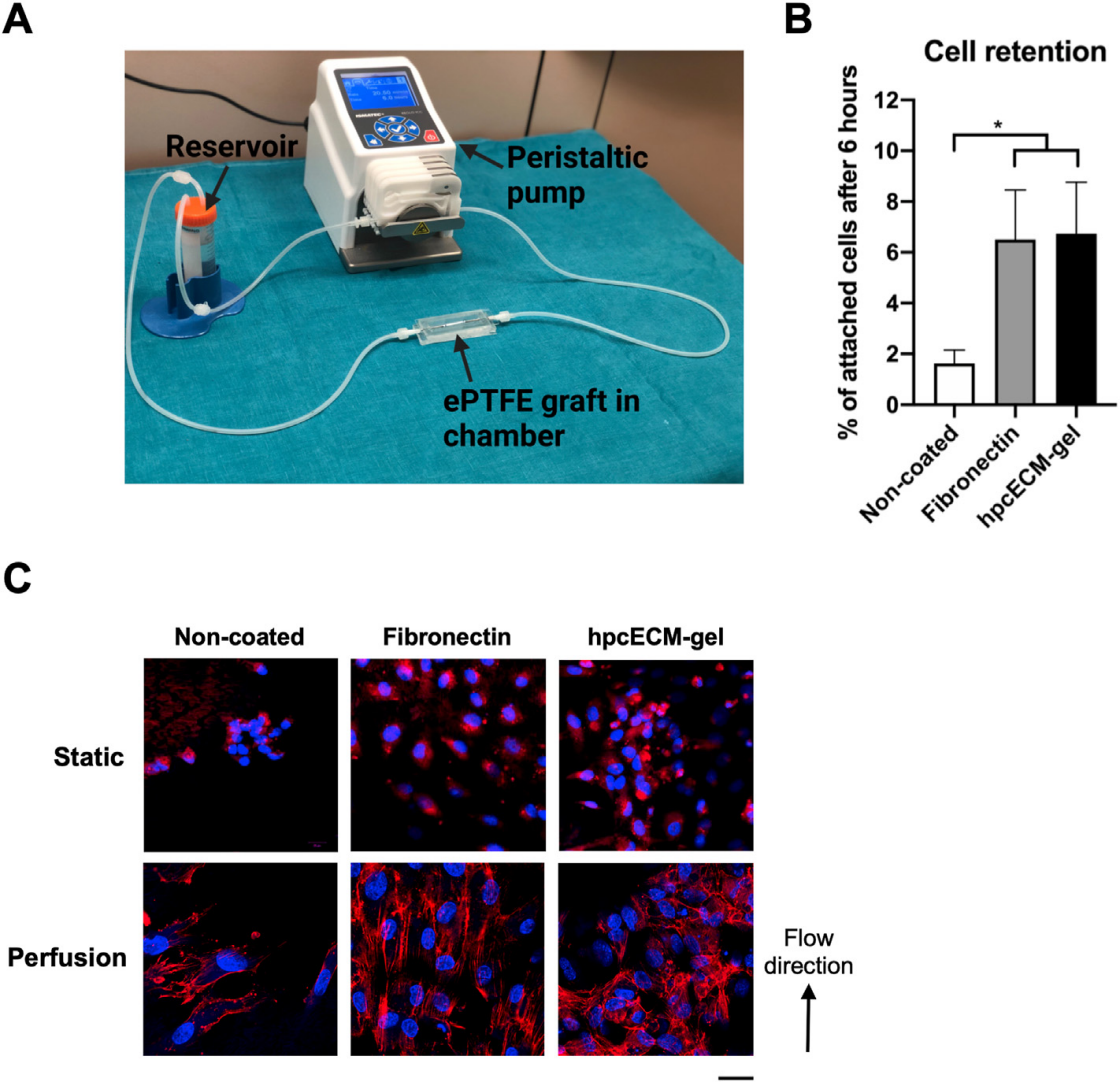
Endothelial cell alignment and retention under flow conditions. The simple perfusion setup consists of a peristaltic pump, the graft subjected to a bioreactor chamber and a reservoir with a 0.22 ​μm filter in the cap (A). Fibronectin, as well as hpcECM-gel enhance endothelial cell retention to flow conditions compared to uncoated surfaces (B). Whereas HUVECs align to flow direction when seeded on fibronectin-coated ePTFE grafts, the morphology remains widely unchanged on non-coated and hpcECM-gel coated conduits (C). Scale bar ​= ​50 ​μm, n ​= ​3.

Moreover, endothelial cell retention to flow was assessed. Fig. 5A represents the simple perfusion set-up used for flow experiments. EGM-2 medium was perfused via a peristaltic pump through grafts subjected to a silicone bioreactor chamber. We evaluated the cell retention to physiological flow conditions. Cell retention to flow was significantly higher in fibronectin (6.5% ​± ​1.96) and hpcECM-gel (6.73% ​± ​2.02) coating conditions compared to non-coated grafts (1.62% ​± ​0.54) (Fig. 5B).

HUVEC alignment to flow direction was evaluated by DAPI/Phalloidin staining. During static cultivation, HUVECs remained rounded without extensive formation of actin filaments. However, under low flow conditions with non-physiological shear stress, HUVECs were aligned in flow direction on fibronectin coated samples whereas in non-coated or hpcECM-gel coated surfaces cells scattered on the surface with no specific alignment (Fig. 5C).

### 3.8 Hemocompatibility assessment

**Fig. 6:**
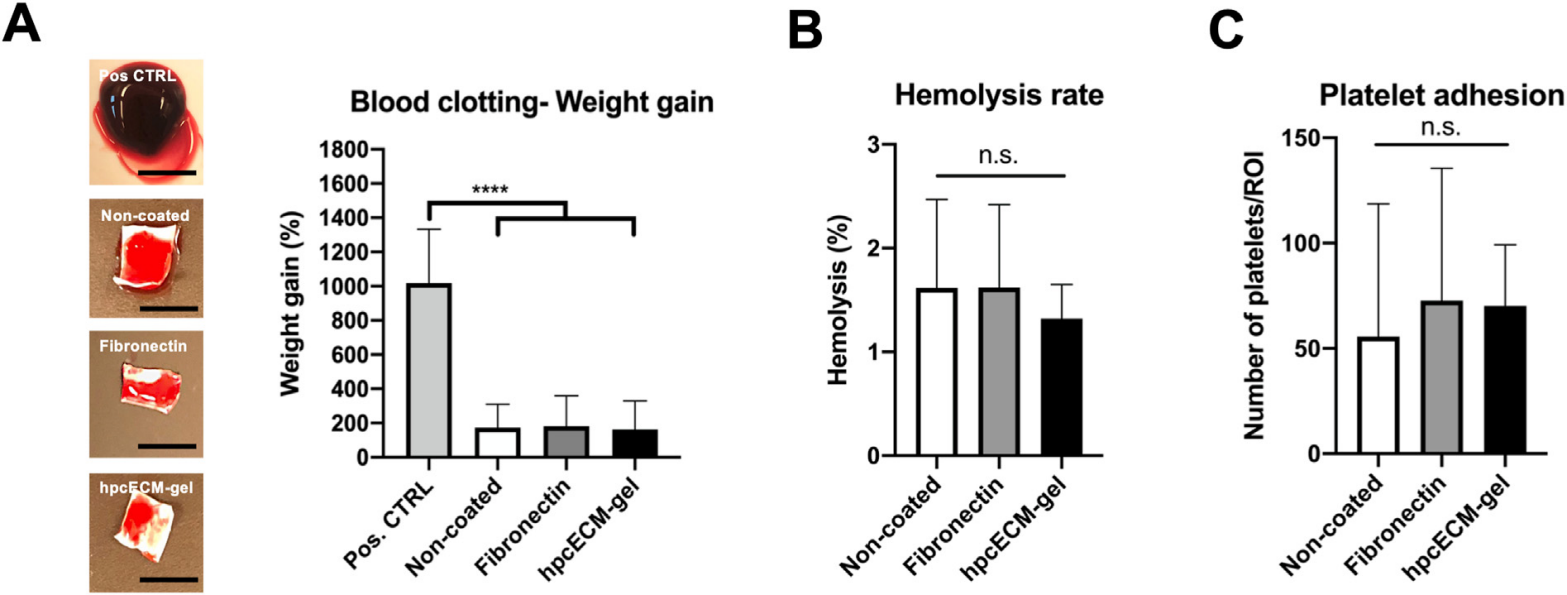
Hemocompatibility assessment of coated surfaces. Neither uncoated, nor fibronectin or hpcECM-gel induced blood clot formation compared to a glass positive control. The images represent respective graft pieces with attached blood clots after incubation time. No significant increase in thrombus formation for any of the observed groups (A). None of the substrates induced hemolysis (B) or increased platelet adhesion (C). n ​= ​6, scale bars: Positive control: 7 ​mm; non-coated, fibronectin and hpcECM-gel: 4 ​mm.

The overall hemocompatibility assessment of the different coatings was carried out via blood clot formation on the graft surface, hemolysis and platelet adhesion. Blood clotting after a static incubation with re-calcified human blood for 1 ​h revealed that neither hpcECM-gel coating (163.04% ​± ​166.51) nor fibronectin (182.2% ​± ​176.95) mediated a change in weight gain due to thrombus formation compared to non-coated surfaces (173.92% ​± ​135.08) (Fig. 6A). Respective images of the graft pieces with attached thrombi demonstrate the quantified blood clot weights (Fig. 6A). All of the groups depicted mean hemolysis rates below 2% (Non-coated: 1.62% ​± ​0.85, Fibronectin: 1.62% ​± ​0.8, hpcECM-gel: 1.32% ​± ​0.33) (Fig. 6B). Furthermore, none of the coatings showed increased platelet adhesion (Non-coated: 55.71 ​± ​62.94, Fibronectin: 72.64 ​± ​62.90, hpcECM-gel: 70.21 ​± ​29.03 ​cells) (Fig. 6C).

## 4 Discussion

Low endothelialization of small diameter ePTFE vascular grafts leads to decreased long-term patency rates compared to autologous vein implants [21]. Therefore, the modification of the ePTFE surface to improve endothelial cell attachment attracted attention recently. A majority of performed studies use growth factor delivery systems, distinct adhesion molecule immobilization or synthetic coatings to enhance endothelial cell attachment and proliferation [[26], [27], [28], [29],[56], [57], [58], [59]]. However, there are still existing drawbacks with these modification methods. For instance, despite outstanding progress in drug delivery systems, this method suffers from some shortcomings such as providing long-term stability, upscaling, optimization of release kinetics and clinical transition [60].

The present study shows the development of a self-made placental chorion-derived extracellular matrix hydrogel and examines its usage as a surface coating to improve the endothelialization of small diameter ePTFE grafts. Our newly established production process results in hpcECM-gels with low DNA residuals and high preservation of structural ECM proteins such as collagen, elastin and proteoglycans as well as fibronectin and laminin. Due to the low immunogenicity of placenta material and the removal of growth factors during the hydrogel processing, we assume that hpcECM-gel could be a potential candidate for small diameter ePTFE graft coating. The idea of using placental material for vascular tissue engineering purposes was already described by Kakavand et al. (2015). In their study, the blood compatibility of human amnion was compared to heparin-coated ePTFE grafts. Platelet adhesion was significantly enhanced on the amniotic membrane. However, it remains unclear whether native amnion might induce immune reaction due to the presence of native cells when they are implanted. Furthermore, endothelial cell attachment (a key element in graft patency), was not investigated [61]. HuBiogel™, another placental hydrogel derived from the amniotic membrane showed promising preliminary results for vascular surface coating [46]. Despite these promising results using amniotic tissue, chorion was used in the current study since significantly higher protein contents can be generated after decellularization (Chorion: 20 ​g; Amnion: 1–4 ​g). Furthermore, the use of a chorionic hydrogel for vascular graft coating has never been investigated before.

Fibronectin was chosen as a positive control in this study since it is a major component of placental hydrogels beside collagen, laminin and hyaluronic acid [62,63]. It was investigated whether the additional presence of laminin and other structural proteins in the hpcECM-gel might lead to an increased endothelialization than pure fibronectin. Furthermore, fibronectin is a well-studied coating material to enhance endothelialization in SDVGs [31], as it contains integrin-binding RGD motifs [64], a key factor for endothelial cell proliferation and adhesion [65,66]. It was previously shown that fibronectin coating enhances endothelial cell attachment to ePTFE surfaces in-vitro [67]. Vohra et al. showed that there is no beneficial effect on endothelial cell adhesion when higher fibronectin concentrations than 50 ​μg/mL are applied [68]. In previous pilot experiments, we evaluated the concentrations used by Vohra et al. and confirmed their findings. Therefore, we chose 50 ​μg/mL fibronectin. In contrast, we found that an optimal cell adhesion was achieved with a hpcECM-gel concentration of 200 ​μg/mL.

Hydrogels are described as highly hydrated polymers (>30% water content per weight) which can be applied as scaffold materials for tissue engineering by maintaining their structural integrity via chemical bonds and physical interactions. A major requirement for ECM hydrogels is the conservation of macromolecular properties to maximize the similarity with natural ECM and consequentially ensuring cytocompatibility [69]. In order to check the suitability of our developed hpcECM-gel as a vascular graft coating material, various biochemical and structural characterizations were carried out in this study. A complete decellularization and removal of DNA is essential during ECM hydrogel production to avoid immunogenic reactions [[70], [71], [72]]. We proved the complete removal of cells and a considerable reduction of DNA after the decellularization process. Gilbert et al. (2009) investigated the DNA content of commercially available ECM scaffold compared to laboratory-produced ones. They showed that a complete removal of DNA could be reached just for two out of nine tested materials. However, the authors state that remaining DNA fragments and even whole cells in dense tissues do not induce immune system responses after implantation [72]. Therefore, we considered our decellularization method a successful way of decellularization for hpcECM-gel. Further assays determining the macromolecular composition showed that collagen was preserved to a great extent during the decellularization process thus providing sufficient structural support for the cell attachment. The results demonstrate that GAGs were present despite the use of Triton X-100 which leads to disruption of the ECM ultrastructure and removal of GAGs [70]. Collagen, laminin, proteoglycans and elastin are the main components of the vascular wall [73]. We hypothesized that hpcECM-gel coating therefore might provide sufficient structural components to enhance endothelial cell attachment on synthetic surfaces.

We further determined the amount of basal lamina proteins, fibronectin and laminin, and found detectable amounts in the hpcECM-gel. Interestingly, both proteins showed high batch-specific concentration. Coating of tissue culture surfaces with hpcECM-gel did not decrease endothelial cell proliferation, viability or migration in preliminary experiments. Additionally, it clearly enhanced HUVECs attachment on tissue culture surfaces after seeding (10 ​min seeding time). HpcECM-gel might therefore be also used as a standard endothelial cell culture substrate in future.

In the current study, SEM and immunofluorescence staining were used to visualize both, fibronectin and hpcECM-gel coating on ePTFE grafts. It was shown before that 40 ​μg/mL fibronectin coating on ePTFE grafts enhanced cell adhesion and the coating was similarly distributed on the surface as in the current study (shown by SEM) [67]. In our study, fibronectin staining revealed successful fibronectin coating of the grafts. HpcECM-gel coating was visualized by immunofluorescence staining and depicted a clearly positive signal for collagen and a faint staining for fibronectin. Similar to our observations, it has been shown in several studies with decellularized tissue hydrogels that the protein fibers are retained despite cryomilling [[74], [75], [76]]. A variety of other spectroscopic techniques, ranging from Atomic Force Microscopy (AFM), Attenuated Total Reflectance Fourier Transform Spectroscopy (ATR-FTIR), X-ray Photoelectron Spectroscopy (XPS) or Time-Of-Flight Secondary Ion Mass Spectroscopy (TOF-SIMS) would help to analyse the coating efficiency in more detail [77].

The proliferation of HUVECs was slightly enhanced on hpcECM-gel compared to the other conditions whereas the viability remained unaffected. Since higher cell numbers were observed in a Live/Dead staining assay, cell viability should have been increased also in the XTT assay. It was shown before that formazan assays are highly susceptible to even small changes of the pH value in the cell culture medium [78]. Therefore, the XTT assay results have to be interpreted with caution.

The adhesion was significantly enhanced on hpcECM-gel compared to both controls. We speculate that this phenomenon is due to the presence of fibronectin and laminin in the hpcECM-gel. Moreover, HUVEC adhesion was enhanced on fibronectin-coated grafts compared to the non-coated control. It was further shown before that the attachment of endothelial colony forming cells (ECFCs) was enhanced on fibronectin-coated synthetic grafts [79]. This is in accordance with our finding showing higher endothelial progenitor cell adhesion on fibronectin compared to non-coated grafts. Similar to HUVECs, endothelial progenitor cell adhesion was significantly higher on hpcECM-gel coating compared to fibronectin-coated grafts. Both, the presence of RGD motifs in fibronectin [64], and their role in cell adhesion [66] was investigated before. However, Le Saux et al. (2011) further identified that topography and RGD ligand density are crucial factors which mediate endothelial cell adhesion [66] thus indicating that not only the amount of RGD in the hpcECM-gel, but also the regions of expression may mediate the enhanced adhesion.

Therefore, integrin expression analyses of HUVECs seeded on coated ePTFE grafts were performed. Expressions of α1, α4 and β5 integrins were not detectable (data not shown). Interestingly, both, fibronectin and hpcECM-gel coating did not mediate a strong increase of integrin expression 10 ​min after seeding compared to non-coated surfaces. Similar expression patterns were observed after 72 ​h incubation time. As described above, cell attachment on fibronectin is mainly mediated via RGD sequence binding to the α5β1 integrin. Thus, an upregulation of α5β1 domain was expected but could not be confirmed. It was shown before that decellularized placental ECM tissues and hydrogels still contain collagen, elastin and GAGs, which are all important cell adhesion proteins [53,80]. Our findings indicate that maybe not RGD sequences alone, but also other factors influence the attachment of cells onto coated surfaces.

The discussed results so far were performed under static conditions. However, it is of outmost importance to observe endothelial cell morphology and retention to physiological flow to evaluate the possible benefit for in-vivo applications. We hypothesized that the hpcECM-gel may favorably influence endothelial cell alignment under pulsatile flow seeding conditions. Cell morphology on hpcECM-gel remained unchanged under slow perfusion (50 ​μL/min) conditions whereas cells on non-coated and fibronectin-coated grafts aligned to flow direction. It was shown before that cells lacking PECAM-1 expression are unable to align to flow direction since PECAM-1 is an important part of the mechanosensory complex which mediates endothelial cell response to flow [81]. We proved that PECAM-1 was significantly higher expressed in the perinuclear region of the cytoplasm when cells were cultured on hpcECM-gel. This expression profile is related to PECAM-1 dimerization and subsequent upregulation of integrin expression which enhances adhesion [82]. However, since no clear upregulation of integrins was detected, the expression pattern of PECAM-1 might be ascribed to a certain level of cell activation as described before [83]. Moreover, the cytoplasmic domain of PECAM-1 is phosphorylated upon mechanical force or cell adhesion to fibronectin and collagen leading to higher cytoplasmic expression of the protein [[84], [85], [86]]. Possibly, the high abundance of phosphorylated cytoplasmic PECAM-1 might inhibit further phosphorylation via flow induction which is necessary for endothelial cell alignment.

To evaluate how hpcECM-gel influences endothelial cell retention under physiological shear stress conditions, coated grafts were subjected to a home-made bioreactor system. High-flow incubation for an hour shows significantly higher cell numbers attached on fibronectin and hpcECM-gel coated grafts compared to non-coated samples.

Endothelial cell activation leads to endothelium dysfunction, resulting in vascular disease. ICAM-1 and VCAM-1 together with E-selectin are major markers for endothelial cell activation [87]. In contrast, PECAM-1 attenuates endothelial cell activation and acts as an important mediator of cell junctional integrity [88]. In our study, all conditions had similar expression of ICAM-1 and VCAM-1. This indicated that hpcECM-gel has not mediated endothelial cell activation cascades. In contrast to PECAM-1, ICAM-1 and VCAM-1 immunostaining did not reveal any specific expression patterns for the observed groups.

Since thrombogenicity remains one of the major reasons for graft failure, hemocompatibility assessment of the grafts with new surface modifications is crucial. Although collagen exposure to blood is known to induce thrombogenicity [89,90], neither blood clotting and hemolysis nor platelet adhesion was enhanced with hpcECM-gel coating which strongly supports an in-vivo use of the coating material.

Infection of prosthetic vascular grafts is a severe complication which leads to mortality rates up to 75% [91]. Placental tissue shows anti-bacterial properties [42]. Therefore, a placental hydrogel coating might help to decrease the risk of a post-surgery bacterial prosthesis infection. Furthermore, the low immunogenicity of placenta tissue [42] may contribute to patient's tolerance of hpECM-gel coated implanted vascular grafts.

## 5 Conclusions

HpcECM-gel depicted a significant enhancement of endothelial cell attachment compared to non-coated and fibronectin-coated ePTFE grafts. We were able to show that hpcECM-gel contains important basal lamina proteins, laminin and fibronectin, which are important for cell adhesion and assure endothelial cell functionality. Furthermore, placental tissue further depicts advantages such as high availability, low immunogenicity and anti-bacterial properties. hpcECM-gel might therefore be a potential alternative to fibronectin as a surface coating material for ePTFE small diameter vascular grafts to increase endothelialization after implantation.

## 6 Limitations

First of all, if clinical application of hpcECM-gel is contemplated, the large-scale manufacturing process will be consistent with GMP guidelines. Moreover, fibrin glue is commercially available and approved for vascular graft coating in clinical applications. Several studies depicted that fibrin glue mediates ePTFE graft endothelialization in clinical long-term settings up to a functional endothelial cell layer [31,32,35,92]. Based on these findings, fibrin glue could have been used in the current study instead of fibronectin. However, cell adherence after 60 ​min was similar on fibrin glue compared to fibronectin during an *in vitro* study [93]. Furthermore, a majority of commercially available fibrin glue products also contain fibronectin [94]. Therefore, we consider the use of fibronectin in the current study as justified. Nevertheless, future studies investigating potential surface coatings for synthetic vascular grafts should consider the clinical success of fibrin glue as a coating substrate and therefore include it as a control.

## Author’s contribution

**Sabrina Rohringer**: Conceptualization, data curation, writing: Original draft. **Karl H. Schneider:** Conceptualization, preliminary data collection, writing: Review and editing. **Gabriela Eder:** Data curation, writing: Review and editing. **Pia Hager:** Data curation, writing: Review and editing. **Marjan Enayati:** Data curation, writing: Review and editing. **Barbara Kapeller:** Investigation, Methodology, writing: Critical reviewing and editing. **Herbert Kiss:** Placenta supply, writing: Review and editing. **Ursula Windberger:** Methodology, data curation, writing: Review and editing. **Bruno K. Podesser:** Writing: Review and editing, funding acquisition. **Helga Bergmeister:** Conceptualization, formal analysis, writing: Original draft, funding acquisition.

## Funding

The study was partly funded by the Ludwig Boltzmann Institute for Cardiovascular Research.

## Data availability

The raw/processed data required to reproduce these findings cannot be shared at this time as the data also forms part of an ongoing study.

## Declaration of competing interest

The authors declare that they have no known competing financial interests or personal relationships that could have appeared to influence the work reported in this paper.
